# Lost in transition: resident and fellow training and experience caring for young adults with chronic conditions in a large United States’ academic medical center

**DOI:** 10.1080/10872981.2019.1605783

**Published:** 2019-05-20

**Authors:** Rebecca E. Sadun, Richard J. Chung, Mclean D. Pollock, Gary R. Maslow

**Affiliations:** a Department of Medicine, Duke University, Durham, NC, USA; b Department of Pediatrics, Duke University, Durham, NC, USA; c Department of Psychiatry, Duke University, Durham, NC, USA

**Keywords:** Adolescents and young adults (AYA), healthcare transition, transfer from pediatric to adult care, transition care skills, graduate medical education, youth with special health care needs (YSHCN)

## Abstract

**Background**: The transition from pediatric to adult healthcare is a vulnerable time for adolescents and young adults (AYA), especially those with chronic conditions. Successful transition requires communication and coordination amongst providers, patients, and families. Unfortunately, multiple studies have demonstrated that the majority of practicing providers do not feel prepared to help AYA patients through health care transition, but little is known about the transition/transfer aptitudes of physician trainees.

**Objectives**: The purpose of this study was to establish the transition/transfer training that residents and fellows from different fields receive – and determine what training factors are associated with increased confidence in core transition/transfer skills.

**Design**: A 20-item electronic survey regarding experiences caring for AYA patients was sent to all 2014–2015 graduate medical education (GME) trainees at our institution.

**Results**: Forty-nine percent (479/985) of trainees responded: 60 pediatric, 387 non-pediatric, and 32 ‘combined’ (e.g., Medicine/Pediatrics or Family Medicine). Trainees from all three categories of programs reported similar exposure to AYA patients with chronic conditions, with a median of 1–3 encounters per month. A quarter of trainees rated themselves as ‘not at all prepared’ to speak with a counterpart provider about a transferring patient, while nearly half of trainees considered themselves ‘not at all prepared’ to speak with a patient and family about transition. Trainee confidence in performing these two skills was strongly predicted by three factors: increased exposure to AYA with chronic conditions, education (training or role modeling) in transition skills, and experience practicing transition skills. Of these, the strongest association with trainee confidence was experience practicing the skills of communicating with other providers (OR = 13.0) or with patients/families (OR = 14.5).

**Conclusion**: Despite at least monthly encounters with AYA with chronic conditions, most residents and fellows have very little experience communicating across the pediatric-to-adult healthcare divide, highlighting training opportunities in graduate medical education.

## Introduction

Approximately 500,000 adolescents and young adults (AYA) with chronic conditions ‘graduate’ from the pediatric medical system each year [1]. For many the transfer to adult care is turbulent, resulting in increased morbidity and mortality [2,3]. In contrast to the act of ‘transfer,’ AYA healthcare ‘transition’ is meant to be a process by which AYA with chronic conditions are prepared to move from ‘child-centered to adult-oriented health-care systems [4].’

Healthcare providers are tasked with helping patients and families navigate this transition [5–8]. Neither pediatricians nor internists feel prepared for this role [9–11], despite the recent development of national guidelines describing transition best practices [5]. AYA with chronic conditions must transition both their primary and specialty care from pediatric to adult settings [12], and the importance of transition has been recognized in myriad clinical settings, from primary care to surgical subspecialties. Despite active discussion about what trainees need to learn in order to be prepared to care for this population [13,14], transition skills are not currently taught in the majority of graduate medical education (GME) programs [15].

The purpose of this study was to investigate how residents and fellows at a large academic medical center are trained in two key AYA transition skills: (1) discussing a transferring patient with a counterpart provider (i.e., a pediatric provider discussing the patient with the adult provider to whom the patient is being transferred, or an adult provider discussing the patient with the pediatric provider from whom the patient is being transferred) and (2) discussing transition and transfer with a young adult patient and his or her family. We hypothesized that didactics and role modeling in transition would be infrequent but would be associated with increased trainee confidence in the ability to provide transition care.

## Methods

### Survey design

**Figure 1.**
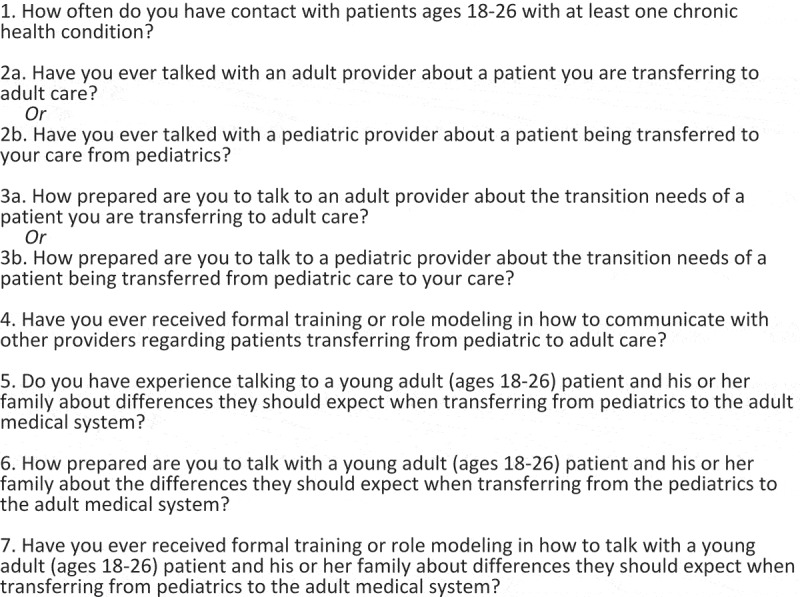
Survey questions.

A 24-item survey was sent by email to all GME trainees at our institution (n = 985) in May 2015. The survey contained four demographics questions, two questions about young adults with disabilities, four questions assessing trainees’ attitudes toward transitioning patients and who bears responsibility for transition and transfer, and seven questions specific to the surveyed institution. In addition, the survey included seven questions pertaining to trainee confidence in two key transition skills, trainee experience performing these two skills, and trainee exposure to formal teaching and role modeling related to these two skills (Figure 1). Prior to their use in this GME-wide survey, the seven questions on confidence, experience, and education were piloted in a Medicine/Pediatrics residency continuity clinic and an interdisciplinary transition clinic with residents from pediatrics, internal medicine, and psychiatry. In both settings, the survey revealed low trainee confidence and minimal trainee exposure to transition education, at baseline. The transition clinic offered residents exposure to and training in many dimensions of transition, and the piloted survey was able to capture statistically significant differences in trainee confidence before compared to after participation in the clinic. Research Electronic Data Capture (REDCap) [16] was used to collect and manage the data.

### Respondants

Training programs were categorized as: (a) pediatric (n = 60 trainees); (b) combined (n = 32) – Pediatric Anesthesia (n = 1), Family Medicine (n = 8), Pediatric Neurology (n = 4), Allergy/Immunology (n = 2), Child/Adolescent Psychiatry (n = 3), Adult/Pediatric Rheumatology fellows (n = 2), and Internal Medicine/Pediatrics (n = 12); or (c) ‘non-pediatric’ (n = 387) – those programs with <10% of training time dedicated to the care of children.

### Primary outcome – self-reported confidence

Trainees were asked to report confidence in two transition skills:

1. Communication with Providers: confidence in discussing a patient being transferred with a counterpart provider.
2. Communication with Patients: confidence in speaking with AYA patients and families about differences between pediatric and adult care systems.

Confidence was rated on a five-point Likert scale, condensed into three categories for analysis: ‘not at all prepared,’ limited confidence (‘beginning to learn’ or ‘developing competence’), and high confidence (‘fully competent’ or ‘highly skilled’).

### Secondary outcomes

Exposure: Trainees were asked to estimate their exposure to AYA with chronic conditions: ‘never,’ ‘1–3 times ever,’ ‘1–3 times per year,’ ‘1–3 times per month,’ or ‘weekly or more.’

Experience: Trainees were asked to estimate the frequency of their communications with transferring patients/families or providers at the time of transfer, using a five-point Likert scale that was then condensed into three categories: never, infrequently (‘1–3 times ever’ or ‘1–3 times per year’), and frequently (‘1–3 times per month’ or ‘weekly or more’).

Education: Trainees were asked about formal training or role-modeling in transition, analyzed as dichotomous variable: never vs some.

Attitudes: Trainees were asked to use a five-point Likert scale to rate their agreement (from ‘strongly agree’ to ‘strongly disagree’) with statements related to AYA transition and transfer, including statements regarding patient readiness at the time of transfer and the physician’s role in preparing patients for transition. For analysis, responses were condensed into three categories: agreement (‘strongly agree’ or ‘agree’), neutral, and disagreement (‘disagree’ or ‘strongly disagree’).

### Recruitment

Trainees were contacted by email with a link to the survey. Trainees were informed that their responses would be de-identified and were offered a $5 gift card for participation.

### Analysis

Data were analyzed using STATA v14 (StataCorp, College Station, TX). Responses were compared using chi-square analysis for a three-way comparison among pediatric, non-pediatric, and combined trainees. Ordinal logistic regression models were constructed to examine the relationship between trainee confidence in performing transition skills and training factors (level of exposure to AYA with chronic conditions, transition education), controlling for trainee factors (age, gender, PGY level, program category). Categorical radiology and pathology trainees were excluded from regression models of confidence because of their limited interactions with patients.

## Results

Forty-nine percent (479/985) of trainees responded, including 60 pediatric trainees, 387 non-pediatric trainees, and 32 combined trainees (Table 1). Nineteen GME programs were represented, with greatest participation from Internal Medicine (n = 131), Surgery (n = 78), and Pediatrics (n = 60).

**Table 1.** Trainee demographics.

	Overall (n = 479)		Pediatric trainees (n = 60)		Adult trainees (n = 387)		Combined trainees (n = 32)	
Age								
25–30	231	(49.8%)	24	(40.7%)	190	(50.9%)	17	(53.1%)
31–36	206	(44.4%)	33	(55.9%)	161	(43.2%)	12	(37.5%)
37–42	19	(4.1%)	0	(0.0%)	17	(4.6%)	2	(6.3%)
43–48	8	(1.7%)	2	(3.4%)	5	(1.3%)	1	(3.1%)
Missing	15		1		14		0	
Gender								
Female	249	(52.0%)	45	(75.0%)	184	(47.6%)	20	(62.5%)
Male	230	(48.0%)	15	(25.0%)	203	(52.5%)	12	(37.5%)
Year in Training								
PGY1	81	(16.9%)	10	(16.7%)	66	(17.1%)	5	(15.6%)
PGY2	92	(19.2%)	10	(16.7%)	77	(19.9%)	5	(15.6%)
PGY3	83	(17.3%)	7	(11.7%)	68	(17.6%)	8	(25.0%)
PGY4	71	(14.8%)	9	(15.0%)	57	(14.7%)	5	(15.6%)
PGY5	74	(15.5%)	12	(20.0%)	54	(14.0%)	8	(25.0%)
PGY6	44	(9.2%)	4	(6.7%)	39	(10.1%)	1	(3.1%)
PGY7 or more	34	(7.1%)	8	(13.3%)	26	(6.7%)	0	(0.0%)
Training Program (residency or sub-specialty fellowship)								
Anesthesiology	45	(9.4%)			44	(11.4%)	1	(3.1%)
Community & Family Medicine	8	(1.7%)					8	(25.0%)
Dermatology	4	(0.8%)			4	(1.0%)		
Emergency Medicine	18	(3.8%)			18	(4.7)		
Medicine	131	(27.4%)			131	(33.9%)		
Neurology	16	(3.3%)			16	(4.1%)		
OB/GYN	29	(6.1%)			29	(7.5%)		
Pathology	7	(1.5%)			7	(1.8%)		
Pediatrics	66	(13.8%)	60	(100.0%)			6	(18.8%)
Psychiatry	22	(4.6%)			19	(4.9%)	3	(9.4%)
Radiology	28	(5.9%)			28	(7.2%)		
Radiology Oncology	5	(1.0%)			5	(1.3%)		
Surgery	78	(16.3%)			78	(20.0%)		
*General Surgery^a^*	*22*	*(4.5%)*			*22*	*(5.7%)*		
*Neurological Surgery*	*6*	*(1.3%)*			*6*	*(1.6%)*		
*Ophthalmology*	*5*	*(1.0%)*			*5*	*(1.3%)*		
*Orthopedic Surgery*	*19*	*(4.0%)*			*19*	*(4.9%)*		
*Otolaryngology*	*5*	*(1.0%)*			*5*	*(1.3%)*		
*Plastic Surgery*	*4*	*(0.8%)*			*4*	*(1.0%)*		
*Thoracic Surgery*	*4*	*(0.8%)*			*4*	*(1.0%)*		
*Urology*	*11*	*(2.3%)*			*11*	*(2.8%)*		
*Other Surgery*	*2*	*(0.4%)*			*2*	*(0.5%)*		
Combined Departments	21	(4.4%)			7	(1.8%)	14	(43.8%)
*Adult & Ped Rheumatology*	*2*	*(0.4%)*					*2*	*(6.3%)*
*Internal Medicine/Pediatrics*	*12*	*(2.6%)*					*12*	*(37.5%)*
*Internal Medicine/Psychiatry*	*7*	*(1.5%)*			*7*	*(1.8%)*		
Global Health Fellowship	1	(0.2%)			1	(0.3%)		

^a^ Italicized data are subsets of the groups under which they are listed; the italicized percentages given represent percentages of all respondents (n = 479) and when summed equal the total (unitalicized) percentage under which they are listed.

### Self-reported confidence

**Figure 2.**
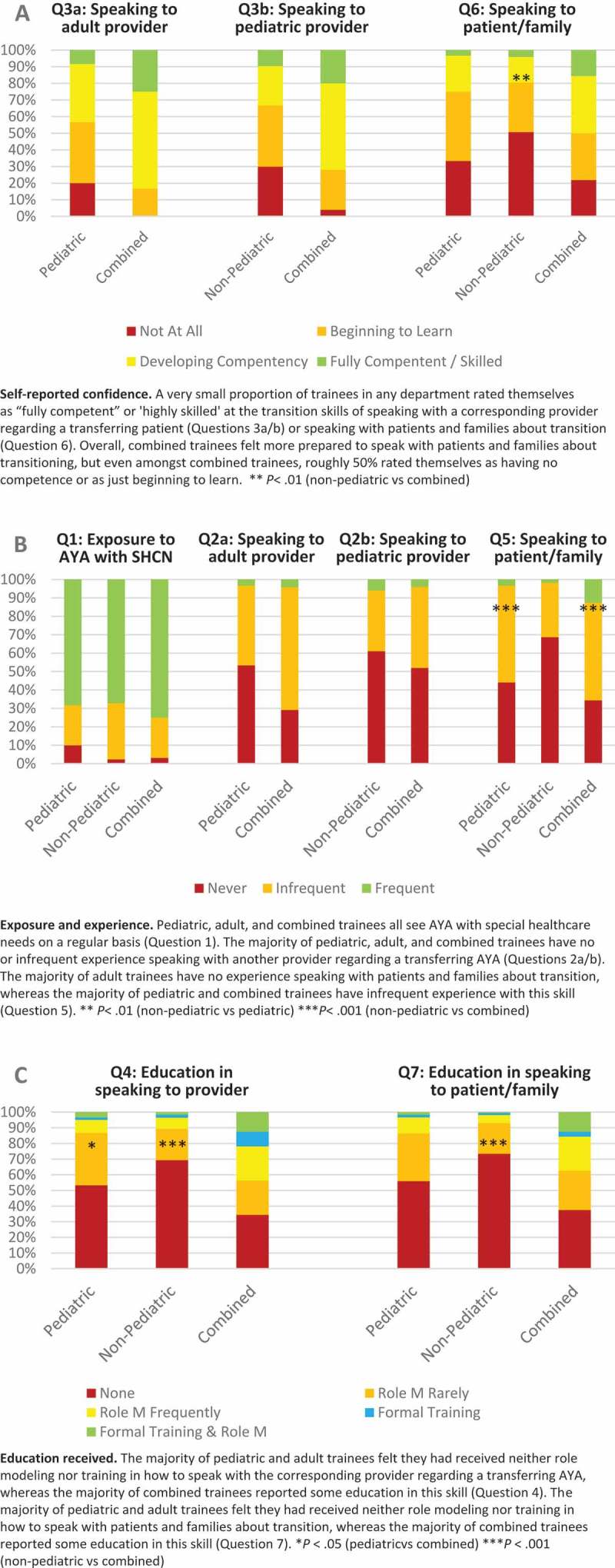
In the formatting I am seeing, this text appears as though it were the end of the discussion section. Please be certain that each of these paragraphs goes beneath the respective part of Figure 2 (A=self-reported confidence, B=exposure and experience, C=education).

With regards to speaking with a counterpart provider about a transferring patient, 30% of non-pediatric respondents, compared with 20% of pediatric respondents, rated themselves as ‘not at all prepared’ (Figure 2(a), Q3a). Fewer than 10% of pediatric or non-pediatric respondents felt ‘fully competent’ or ‘highly skilled’ at discussing a transitioning patient with their counterpart provider, compared with 20+% of the combined trainees. Among advanced trainees (PGY-3 and beyond), more than a third of pediatric trainees and over half of non-pediatric trainees reported that they were ‘not at all prepared’ or were only ‘beginning to learn’ to speak with counterpart adult providers (data not shown).

Only 3% of pediatric trainees, 4% of non-pediatric trainees, and 16% of combined trainees reported being ‘fully competent’ or ‘highly skilled’ at educating patients and families about what to expect during transfer (Figure 2(a), Q6); in contrast, 33% of pediatric trainees, 51% of non-pediatric trainees, and 22% of combined trainees felt not at all prepared (*P* < .01). Self-reported confidence increased somewhat with level of training, but over 40% of PGY3+ trainees reported feeling ‘not at all prepared’ to perform this skill (data not shown).

### Exposure and experience

Trainee exposure to AYA patients with chronic conditions was similar among all trainees, with mean, median, and mode being ‘1–3 times per month’ (Figure 2(b), Q1) and a very similar overall distribution. Fifty-three percent of pediatric trainees and 61% of non-pediatric trainees reported no experience in communicating with other providers about AYA patients transferring out of pediatrics (Figure 2(b), Q2a and Q2b). No experience communicating with patients and their families was reported by 69% of non-pediatric trainees, 44% of pediatric trainees, and 34% of combined trainees (Figure 2(b), Q5), with non-pediatric trainee experience being statistically significantly lower than trainees with pediatric or combined training (*P* < .001 for non-pediatric vs combined; *P* < .01 for non-pediatric vs pediatric).

### Education

Regarding communicating with other providers, 53% of pediatric trainees and 69% of non-pediatric trainees reported receiving neither training nor role modeling (Figure 2(c), Q4). For combined trainees, however, 34% reported receiving neither training nor role modeling, which was statistically significantly different than pediatric (*P* < .05) or non-pediatric (*P* < .001) trainees.

With regards to speaking with patients and families about transition, 56% of pediatric trainees, 73% of non-pediatric trainees, and 38% of combined trainees reported receiving neither training nor role modeling (Figure 2(c), Q7). Combined trainees again reported more training than non-pediatric trainees (*P* < .001), although only 13% of combined trainees reported receiving both training and role modeling. Importantly, PGY3 and beyond trainees were not more likely to report receiving training or role modeling.

### Multivariable modeling of confidence

Multivariate models for self-reported confidence with (1) communication with other providers and (2) communication with patients and families identified three factors that strongly predicted trainee confidence: increased exposure to AYA with chronic conditions, receiving role modeling and/or training, and increased experience/practice (Table 2). Experience practicing the core skills was the most significant variable in trainees’ reported confidence, demonstrating a dose-response: Compared to trainees reporting no experience, trainees reporting infrequent experience had 4.2 and 8.68 times the odds, and those reporting frequent experience practicing transition skills had 13 times and 14.5 times the odds, respectively, of high confidence in their ability to speak with a provider or with a patient and family about transition (*P* < .001).

**Table 2.** Adjusted^a^ odds ratios for multivariate modeling of trainee confidence in transition skills.

	Prepared to talk to counterpart provider^b^			Prepared to talk to patient/family^c^		
	*Odds Ratio*	*95% CI*	*P value*	*Odds Ratio*	*95% CI*	*P value*
**Exposure (Q1)**						
*Comparison: none*	*1.0 (ref)*					
Infrequent exposure	4.7	1.4–16.2	.014	11.5	2.0–67.2	.007
Frequent exposure	7.2	2.1–24.6	.002	21.3	3.7–121.2	.001
**Experience (Q2/5)**						
*Comparison: none*	*1.0 (ref)*					
Infrequent experience	4.2	2.8–6.3	<.001	8.6	5.4–13.5	<.001
Frequent experience	13.0	5.6–30.4	<.001	14.5	4.6–45.6	<.001
**Education (Q4/7)**						
*Comparison: none*	*1.0 (ref)*					
Role modeling	2.0	1.2–3.4	.008	3.5	1.8–6.6	<.001
Formal training	5.3	1.9–14.5	.001	4.6	1.2–17.0	.024

^a^**Adjusted for**: age, gender, PGY level, and program type

^b^**Question 3**: How prepared are you to talk to an [adult/pediatric] provider about the transition needs of a patient [you are transferring/being transferred to you]?

^c^**Question 6**: How prepared are you to talk with a young adult (ages 18–26) patient and his or her family about the differences they should expect when transferring from the pediatrics to the adult medical system?

### Attitudes

Whereas 89% of trainees agreed with the statement, ‘Physicians have a responsibility to prepare young adults to independently manage their health condition(s),’ only 7.5% of trainees agreed with the statement, ‘Young adults transferred from a pediatric practice are usually prepared to navigate the adult medical system.’

## Discussion

In this study, trainees in 19 specialties reported caring for AYA with chronic conditions an average of 1–3 times monthly. Trainees nevertheless reported minimal confidence in their ability to perform two key transition care skills, and very few trainees reported formal instruction or role-modeling in how to speak with patients, families, or providers about transition. In regression analyses, formal training, role modeling, and practice performing transition skills were all robust predictors of trainee self-reported confidence. Combined trainees reported the most confidence with transition skills; however, the majority of combined trainees still reported little to no training or role modeling and low levels of confidence. Furthermore, many trainees at or beyond their third year of training believed they had not even begun to learn these skills; given that some residencies including primary care residencies like pediatrics, internal medicine, and family medicine are only three years in length, this raises concern that advanced and even graduating trainees frequently lack confidence in the core skills of transition.

The vast majority of trainees felt AYA with chronic conditions are usually unprepared for transfer and agreed that physicians are responsible for helping AYA transition. Trainees’ lack of experience engaging with transition skills may be attributable to lack of confidence and therefore may be able to be addressed through educational interventions.

This study has several limitations, including being a single institution survey, which raises concern for generalizability, and having a survey response rate of 50%, which leaves opportunity for response bias. In addition, while the survey was piloted in two clinics prior to this study, it should be noted that it was not validated for all clinical settings. Conversely, a strength of this study is that it captures responses from trainees in a diverse array of fields, whereas most studies of transition education have been limited to primary care trainees or practicing physicians in a single field. Furthermore, to address generalizability concerns, given the single institution nature of this study, we were able to verify that fellows, more than half of whom completed residencies at outside institutions, are not different with regards to their confidence, experience, or education in the realm of transition, when compared with PGY-similar residents from our institution.

This study confirms that many trainees, across a broad range of specialties, care for AYA with chronic conditions, though few are taught how to ‘handoff’ or ‘receive’ AYA patients. Even PGY3+ trainees often do not feel prepared to support AYA through transition and transfer. This is concerning given the central role that providers play in preventing poor outcomes for transitioning AYA [17].

An estimated 15% of adolescents are identified as having special healthcare needs [18]. While many of these patients have a primary care medical home, some do not, and most receive a substantial percentage of their care from subspecialists. Accordingly, an increasing number of fields are recognizing the subspecialist’s role in ensuring continuity of care for transitioning young adults [10], with pediatric surgery [19] and peri-operative anesthesia [20] being amongst the most recent subspecialties to call for concerted transition efforts within their fields as a necessary complement to primary care transition.

To effectively serve in this capacity, however, physicians must develop confidence and competence in the skills required to deliver high quality transition care, learning either how to prepare young adults for transition (if the trainee is in a pediatrics-based field), or how to receive them with a ‘soft landing’ into adult care after transition (if the trainee is in an adult-predominant medical field); combined trainees need to be proficient in both of these skills. As of 2018, training in transition and transfer skills had not become a residency requirement in Pediatrics, Internal Medicine, Medicine/Pediatrics, or Family Medicine [21].

The literature is beginning to include pilot curricula for trainees [22, 23], including creative ways for pediatric and internal medicine trainees to learn from each other’s’ expertise [22]. There are also many tools and resources offered on the National Center for Healthcare Transition’s website, GotTransition.org [24], and training on the use of these tools would itself constitute a curriculum in clinically-relevant transition and transfer skills.

Recently, international expert opinion was sought from 56 medical professionals with expertise in healthcare transition, using a modified Delphi process to identify five educational goals with 32 associated objectives for resident trainees in primary care fields [25]. The 32 objectives included eight objectives related to understanding the healthcare transition process, of which five were skill-based objectives:

- perform a complete history and physical in an AYA with physical and/or intellectual disabilities
- communicate and coordinate effectively around handoffs in healthcare transition between pediatric and adult providers
- demonstrate the ability to work as an effective member of an interprofessional team to support transitioning AYA
- develop and update transition care plans, addressing patients’ decision-making status and self-management skills
- apply effective coding and billing practice for healthcare transition services, and use a transfer package, including a readiness assessment, medical summary, emergency care plan, and any needed legal documents

In addition, they identified four objectives pertaining to understanding the implications of insurance eligibility and social services as they relate to the transitioning population, six objectives related to using systems-based practice to improve patient care and policies for AYA with special healthcare needs, and 16 objectives related to the developmental, psychosocial, and educational/vocational needs of AYA with chronic conditions.

As training programs and medical education accrediting bodies begin to think about the skills physicians need to learn to provide safe and effective medical care to transitioning AYA, this study helps establish a baseline in trainees’ confidence in core transition skills. In addition, this study highlights that it is critical for curricula to provide trainees with opportunities for skills-based practice, as the practice of core transition skills was most highly associated with growth in trainees’ self-assessment of proficiency.

Indeed, in order for physicians to exercise transition best practices, trainees must learn about them, see them role modeled, and have opportunity to practice them under guidance.
